# Development of a broad-host synthetic biology toolbox for ralstonia eutropha and its application to engineering hydrocarbon biofuel production

**DOI:** 10.1186/1475-2859-12-107

**Published:** 2013-11-13

**Authors:** Changhao Bi, Peter Su, Jana Müller, Yi-Chun Yeh, Swapnil R Chhabra, Harry R Beller, Steven W Singer, Nathan J Hillson

**Affiliations:** 1 Physical Biosciences Division, Lawrence Berkeley National Laboratory, Berkeley, CA 94720, USA; 2 Present address: Tianjin Institute of Biotechnology, Chinese Academy of Sciences, Tianjin, China; 3 Department of Chemical & Biomolecular Engineering, University of California, Berkeley, CA 94720, USA; 4 National Taiwan Normal University, Taipei, Taiwan; 5 Earth Sciences Division, Lawrence Berkeley National Laboratory, Berkeley, CA 94720, USA

**Keywords:** Broad-host, Synthetic biology, *Ralstonia eutropha*, Hydrocarbon, Chemolithoautotroph

## Abstract

**Background:**

The chemoautotrophic bacterium *Ralstonia eutropha* can utilize H_2_/CO_2_ for growth under aerobic conditions. While this microbial host has great potential to be engineered to produce desired compounds (beyond polyhydroxybutyrate) directly from CO_2_, little work has been done to develop genetic part libraries to enable such endeavors.

**Results:**

We report the development of a toolbox for the metabolic engineering of *Ralstonia eutropha* H16. We have constructed a set of broad-host-range plasmids bearing a variety of origins of replication, promoters, 5’ mRNA stem-loop structures, and ribosomal binding sites. Specifically, we analyzed the origins of replication pCM62 (IncP), pBBR1, pKT (IncQ), and their variants. We tested the promoters P_BAD_, T7, P_xyls/PM_, P_lacUV5_, and variants thereof for inducible expression. We also evaluated a T7 mRNA stem-loop structure sequence and compared a set of ribosomal binding site (RBS) sequences derived from *Escherichia coli*, *R. eutropha*, and a computational RBS design tool. Finally, we employed the toolbox to optimize hydrocarbon production in *R. eutropha* and demonstrated a 6-fold titer improvement using the appropriate combination of parts.

**Conclusion:**

We constructed and evaluated a versatile synthetic biology toolbox for *Ralstonia eutropha* metabolic engineering that could apply to other microbial hosts as well.

## Background

Chemoautotrophic “Knallgas” bacteria can utilize H_2_/CO_2_ for growth under aerobic conditions, and have great potential to directly produce liquid fuels from CO_2_ and/or syngas [1,2]. *Ralstonia eutropha* (*R. eutropha*), the model bacterium of this class, can grow to very high cell densities (>200 g/L) [3]. Under nutrient limitation, *R. eutropha* directs most of its carbon flux to the synthesis of polyhydroxybutyrate (PHB), a biopolymeric compound stored in granules. Under autotrophic growth conditions with H_2_/CO_2_, *R. eutropha* has been reported to synthesize 61 g/L of PHB (representing ~70% of total cell weight) in 40 h [4]. With random mutagenesis and relatively simple engineering, PHB and related polyhydroxyalkanoate polymers have been produced in *R. eutropha* on industrial scales [3].

While *R. eutropha* has great potential to be engineered to produce desired compounds (beyond PHB) directly from CO_2_, little work has been done to develop genetic part libraries to enable such endeavors. Although suicide vectors have been used to generate in-frame deletions and point mutations in *R. eutropha*[5], and previously reported broad-host range expression systems [6] may be transferable to *R. eutropha*, to date, the only established inducible expression system for *R. eutropha* has been a pBBR1-derived vector with a P_BAD_ promoter [7]. Here, we have initiated the development of a synthetic biology toolbox to enable complex metabolic engineering applications in *R. eutropha* H16. We evaluated a variety of vectors, promoters, 5’ mRNA stem-loop sequences, and ribosomal binding sites (RBSs), and rationally mutated and engineered these genetic components to improve and diversify their function in *R. eutropha*. We then applied the resulting toolbox to engineer and optimize a hydrocarbon production pathway. Taken together, this work develops and demonstrates the engineering utility of a plasmid-based toolbox for *R. eutropha*.

## Results

### Broad-host vector evaluation and engineering

**Figure 1:**
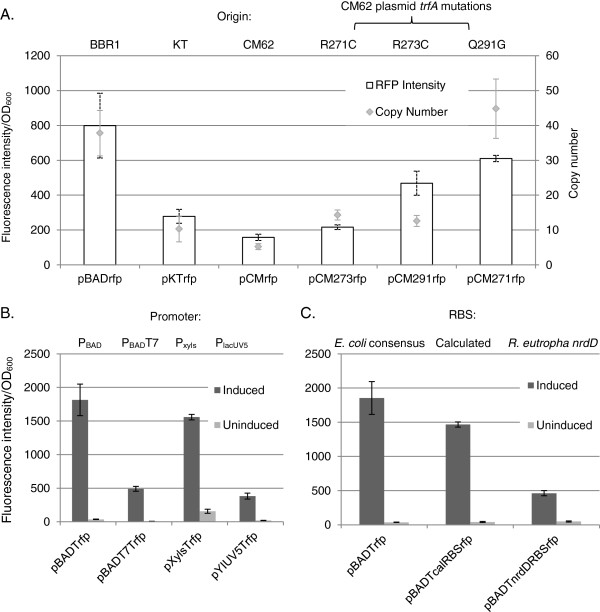
**Plasmid expression vector RFP fluorescence intensities and copy numbers. (A)** Induced RFP fluorescence intensity/OD_600_ (dark bars) and plasmid copy numbers (square dots) for the various origins of replication. **(B, C)** Induced (dark bars) and uninduced (light bars) RFP fluorescence intensity/OD_600_ for the various **(B)** promoters and **(C)** RBS sequences.

Three broad-host-range plasmid vectors were selected as starting points for the construction of new plasmid-based expression systems for *R. eutropha*: 1) pCM62, a low-copy-number plasmid within the IncP incompatibility group [8]; 2) pBBR1MCS, a medium-copy-number plasmid [9]; and 3) pKT230, a high-copy-number plasmid within the IncQ group containing the RSF1010 origin [10]. The kanamycin-resistance selection marker within pKT230 was replaced with a chloramphenicol-resistance marker to enable co-selection with pCM62 and pBBR1MCS-derivative plasmids. An inducible *rfp* expression cassette containing a P_BAD_ promoter [7], an *E. coli* consensus RBS, *rfp*, and a double terminator was incorporated into all three plasmid types. While none of the resulting plasmids were successfully electroporated into *R. eutropha*, they were all successfully transconjugated. As shown in Figure 1A, pBADrfp (the pBBR1MCS-derivative, see Table 1) provided the highest induced RFP expression level, while pKTrfp and pCMrfp had lower expression levels. Plasmid pBADrfp (BBR1 origin, kanamycin resistance) co-propagated stably with pKTrfp (KT origin, chloramphenicol resistance) or pCMrfp (CM62 origin, tetracycline resistance) in *R. eutropha* (data not shown).

**Table 1:** Strains and plasmids used in this study

**Strain**	**Description**	**Reference**
*R. eutropha* H16	*R. eutropha* wildtype strain currently classified as *Cuparividus necator*	ATCC 17669
*R. eutropha* H16 ∆2303	H16 Δ(H16_A0459-0464, H16_A1526-1531); Δbeta ox; mutant is deficient in native-oxidation	[11]
*E. coli* DH10B	F' *proA*^+^*B*^+^*lacI*^*q*^ ∆*lacZM15*/ *fhuA2* ∆(*lac*-*proAB*) *glnV galR* (*zgb*-*210*::*Tn10*) Tet^S^*endA1 thi*-*1* ∆(*hsdS*-*mcrB*)*5*	NEB
*E. coli* S17	*E. coli* host strain for transconjugation	[12]
**Plasmid**	**Description**	**Reference**
pCM62	Broad host-range plasmid IncP group; *amp*^*R*^, *tet*^*R*^	[8]
pBBR1MCS	Broad host-range plasmid compatible with IncQ, IncP, IncW, and colE1; *kan*^*R*^	[9]
pKT230	Broad host-range plasmid IncQ group; *kan*^*R*^	[10]
pBADrfp	pBBR1MCS derivative; P_BAD__*rfp*	[13]
pBbE8c-RFP	colE1; P_BAD__*rfp*; cm^*R*^	[14]
pBbA8a-RFP	p15a; P_BAD__*rfp*; amp^*R*^	[14]
pKTrfp	pKT230 derivative; P_BAD__*rfp*; *cm*^*R*^	This study
pCMrfp	pCM62 derivative; P_BAD__*rfp*; *amp*^*R*^, *tet*^*R*^	This study
pCM271rfp	pCMrfp with TrfA R271C mutation	This study
pCM273rfp	pCMrfp with TrfA R273C mutation	This study
pCM291rfp	pCMrfp with TrfA Q291G mutation	This study
pBADTrfp	pBADrfp derivative; P_BAD__T7 stem-loop_*rfp*	This study
pXylsTrfp	pBADTrfp derivative; P_xyls/PM__T7 stem-loop_*rfp*	This study
pUV5Trfp	pBADTrfp derivative; P_lacUV5__T7 stem-loop_*rfp*	This study
pIUV5Trfp	pBADTrfp derivative; *lacI*^*q*^; P_lacUV5__T7 stem-loop_*rfp*	This study
pTetTrfp	pBADTrfp derivative; P_tet__T7 stem-loop_*rfp*	This study
pProErfp	pBADTrfp derivative; P_proE__ *rfp*	This study
pProSrfp	pBADTrfp derivative; P_proS__ *rfp*	This study
pBADT7Trfp	pBADTrfp derivative; P_BAD__T7 polymerase; PT7_T7 stem-loop_*rfp*	This study
pYIUV5Trfp	pBADTrfp derivative; *lacY lacI*^*q*^; P_lacUV5__T7 stem-loop_*rfp*	This study
pBADTcalRBSrfp	pBADTrfp derivative; P_BAD__T7 stem-loop_calRBS_rfp__*rfp*	This study
pBADTnrdDRBSrfp	pBADTrfp derivative; P_BAD__T7 stem-loop_nrdDRBS_*rfp*	This study
pKTTrfp	pKT230 derivative; P_BAD__ T7 stem-loop_*rfp*; *cm*^*R*^	This study
pCMTrfp	pCM62 derivative; P_BAD__ T7 stem-loop_*rfp*	This study
pCM271Trfp	pCM62 derivative; pCMTrfp with TrfA R271C mutation	This study
pCM271TcalRBSrfp	pCM271rfp derivative; P_BAD__T7 stem-loop_calRBS_rfp__*rfp*	This study
pBADTHC	pBADrfpT derivative; P_BAD__T7 stem-loop_*aar*_*adc*	This study
pKTTHC	pKTrfp derivative; P_BAD__T7 stem-loop_*aar*_*adc*	This study
pCMTHC	pCM62 derivative; P_BAD__T7 stem-loop_*aar*_*adc*	This study
pCM271THC	pCM271rfp derivative; P_BAD__T7 stem-loop_*aar*_*adc*	This study
pBADHC	pBADrfp derivative; P_BAD__*aar*_*adc*	This study
pXylsTHC	pBADrfp derivative; P_xyls/PM__T7 stem-loop_*aar*_*adc*	This study
pYIUV5THC	pYIUV5Trfp derivative; P_lacUV5__T7 stem-loop_*aar*_*adc*	This study
pBADTcalRBSHC	pBADTHC derivative; P_BAD__T7 stem-loop_calRBS_aar__*aar*_calRBS_adc__*adc*	This study
pBADTnrdDRBSHC	pBADTHC derivative; P_BAD__T7 stem-loop_nrdDRBS_*aar*_ nrdDRBS_*adc*	This study
pCM271TcalRBSHC	pCM271rfp derivative; P_BAD__T7 stem-loop_ calRBS_aar__*aar*_calRBS_adc__*adc*	This study

To increase the copy number of the pCMrfp plasmid, previously reported site-directed mutations were made to the *trfA* gene [15]. While putatively high-copy-number pCMrfp mutants (TrfA positions 251, 254 and 234) were not successfully transconjugated and established in *R. eutropha*, possibly because high-copy-number plasmids are not well tolerated in *R. eutropha*[16], medium-copy-number pCMrfp mutants (TrfA R271C, R273C and Q291G) were established. The mutant pCMrfp plasmids pCM271rfp, pCM273rfp, and pCM291rfp were measured to have higher RFP expression levels than pCMrfp (Figure 1A). To determine the absolute copy numbers of the pCMrfp plasmid variants, qPCR was performed using *R. eutropha* colonies as the source of the template (Figure 1A). pCM271rfp was determined to have the highest copy number (44.8 ± 8.5 copies per cell) among the pCMrfp variants. pCM273rfp and pCM291rfp both had higher copy numbers than pCMrfp.

### T7 stem-loop structure evaluation

A T7 stem-loop structure [17] was inserted upstream of the RBS of the *rfp* gene on plasmid pBADrfp, yielding pBADTrfp. Introducing the T7 stem-loop structure into pBADTrfp increased RFP expression (1814 ± 236 RFP intensity/OD, Figure 1B) by approximately 2-fold over pBADrfp levels (798 ± 185 RFP intensity/OD) (Figure 1A).

### Inducible promoter system evaluation and engineering

In addition to P_BAD_, several other inducible promoter systems were evaluated in *R. eutropha*. Various repressor or activator genes along with their respective operators and promoters were inserted into pBADTrfp, replacing *araC*/P_BAD_. As shown in Figure 1B, the P_BAD_ (pBADTrfp) and P_xyls/PM_ (pXylsTrfp) promoter systems provided the highest RFP expression upon induction. This is the first demonstration that the P_xyls/PM_ promoter system is functional in *R. eutropha*. The T7 promoter controlled by P_BAD_-induced T7 polymerase (pBADT7Trfp), although only providing modest RFP expression upon induction, had very little expression in the absence of induction. P_lacUV5_ (pUV5Trfp and pIUV5Trfp), P_tet_ (pTetTrfp), and P_pro_ (pProErfp and pProSrfp) systems did not show inducible expression in *R. eutropha* (Additional file 1: Figure S1, and data not shown). The P_lac_/lacI system has been reported previously not to be functional in *R. eutropha*[18]. Genomic sequence comparison between *R. eutropha* H16 and *E. coli* revealed that *R. eutropha* lacks the galactose permease gene *lacY*. This permease facilitates the transportation of lactose as well as the P_lac_ inducer IPTG into *E. coli*[19]. A *lacY* gene codon-optimized for *R. eutropha* expressed from a constitutive promoter was incorporated into pIUV5Trfp, yielding pYIUV5Trfp. As shown in Figure 1B and Additional file 1: Figure S1, the incorporation of the *lacY* gene into pYIUV5Trfp enabled the IPTG-inducible expression of RFP from P_lacUV5_, although the expression level is low compared to those of P_BAD_ and P_xyls/PM_.

Cross-induction perturbation assays were performed to test if the chemical inducers L-arabinose (P_BAD_), *m*-toluic acid (P_xyls/PM_), and IPTG (P_lacUV5_) affect the performance of their non-cognate promoter systems (Table 2). For the most part, the three chemical inducers did not significantly perturb their non-cognate promoter systems. For example, the induction of Pxyls/PM by 1 mM *m*-toluic acid retained 95.4% and 98.0% of normal levels, respectively, when 1 mM IPTG or 0.1% L-arabinose were added. An important exception is that 1 mM *m*-toluic acid negatively impacted the induction of P_BAD_ by 0.1% L-arabinose to about 60% of normal levels. However, when the *m*-toluic acid concentration was reduced from 1 mM to 0.5 mM, the induction of P_BAD_ by 0.1% L-arabinose remained at 91.7 ± 1.6% of normal levels.

**Table 2:** **Promoter cross**-**induction test**

**Promoter**	**Non-cognate inducer added**		
	**L-arabinose**	***m*-toluic acid**	**IPTG**
PBAD	(100%)^a^	61.5 ± 3.9%	105.0 ± 28.8%
Pxyls/PM	98.0 ± 2.7%	(100%)^a^	95.4 ± 9%
PlacUV5	97.4 ± 3.3%	91.0 ± 0.24%	(100%)^a^

^a^Cognate inducer alone.

Observed florescence intensity relative to cognate inducer alone.

### Ribosomal binding site sequence evaluation

Three RBS sequences were evaluated to compare their translation initiation efficiencies in *R. eutropha*: 1) an *E. coli* consensus RBS sequence (pBADTrfp), 2) a RBS calculator [20] designed RBS sequence (pBADTcalRBSrfp), and 3) the *R. eutropha nrdD* RBS sequence (pBADTnrdDRBSrfp). RBS calculator parameters were specified towards designing a strong RBS sequence for *R. eutropha*, with the setting at “max”, provided pBADTrfp RBS region context. The *E. coli* consensus RBS provided the highest RFP expression levels (Figure 1C), while the computationally designed RBS provided medium to high RFP expression, and the native *R. eutropha nrdD* RBS provided the lowest RFP expression levels.

### Applying the toolbox to hydrocarbon production optimization

**Figure 2:**
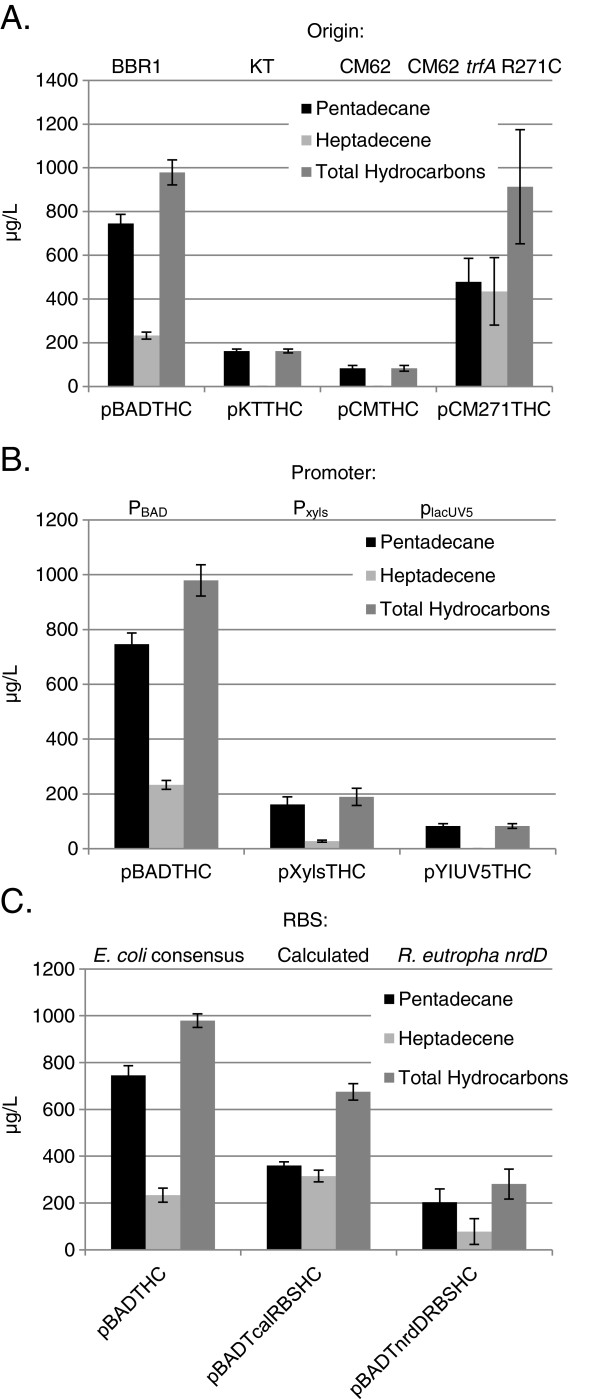
Hydrocarbon titers for the various (A) origins of replication, (B) promoters, and (C) RBS sequences; (dark bars) pentadecane, (light bars) heptadecene, and (grey bars) combined.

**Figure 3:**
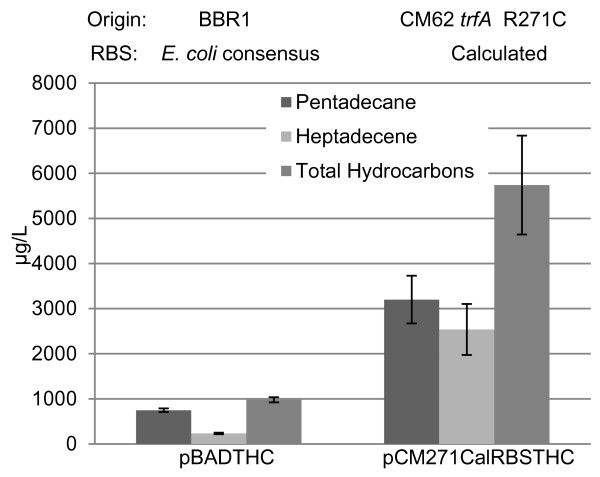
Hydrocarbon titers for the two highest producing strains.

**Figure 4:**
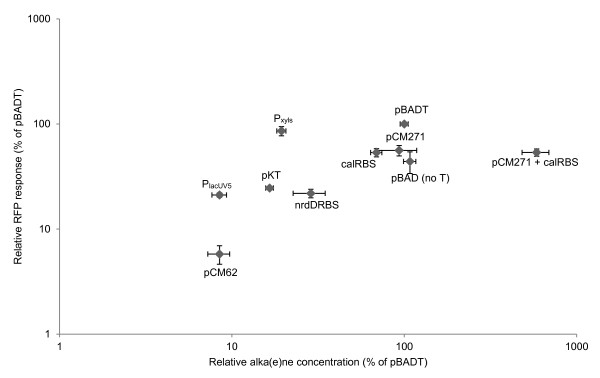
**RFP expression levels and hydrocarbon production titers.** Values normalized to reference plasmids pBADTrfp and pBADTHC. pCM62: pCMTrfp and pCMTHC. P_lacUV5_: pYIUV5Trfp and pYIUV5THC. pKT: pKTTrfp and pKTTHC. P_xyls_: pXylsTrfp and pXylsTHC. nrdDRBS: pBADTnrdDRBSrfp and pBADTnrdDRBSHC. calRBS: pBADTcalRBSrfp and pBADTcalRBSHC. pCM271: pCM271Trfp and pCM271THC. pBADT: pBADTrfp and pBADTHC. pBAD (no T): pBADrfp and pBADHC. pCM271 + calRBS: pCM271TcalRBSrfp and pCM271TcalRBSHC.

The synthetic biology toolbox was iteratively applied to optimize hydrocarbon production in *R. eutropha*. Genes encoding acyl-ACP reductase (*aar*) and aldehyde decarbonylase (*adc*) [21] were codon optimized for *R. eutropha* and synthesized (GenScript). These two synthesized genes were incorporated as an operon into the *R. eutropha* expression vectors developed above. The first set of constructed vectors (pBADTHC, pKTTHC, pCMTHC, and pCM271THC) was designed to determine the impact of plasmid origin of replication on hydrocarbon product titer (Figure 2A). Independent of the origin of replication, expressing the *aar*-*adc* hydrocarbon pathway in *R. eutropha* H16 resulted predominantly in the production of pentadecane (from palmitic acid) and heptadecene (likely from oleic acid [1]). The pBADTHC plasmid (pBBR1 origin) achieved the highest combined (pentadecane + heptadecene) titer, whereas the pCMTHC plasmid (pCM62 origin) produced the lowest. The pCM271THC plasmid (mutant pCM271 origin) was able to achieve a combined hydrocarbon titer comparable to that of pBADTHC, albeit with a more balanced pentadecane:heptadecene ratio. Removing the T7 stem-loop structure from pBADTHC, yielding plasmid pBADHC, did not significantly affect hydrocarbon titer, with the combined hydrocarbon for both reaching approximately 1000 μg/L (Additional file 1: Figure S2). The next set of constructed vectors (pXylsTHC and pYIUV5THC) was designed to determine the impact of the promoter on hydrocarbon product titer (Figure 2B). Of the three promoters tested, P_BAD_ achieved the highest levels of hydrocarbon production, while P_xyls/PM_ and P_lacUV5_ only achieved low hydrocarbon titers (Figure 2B). The final set of constructed vectors (pBADTcalRBSHC and pBADTnrdDRBSHC) was designed to determine the impact of the RBS sequence on hydrocarbon product titer (Figure 2C). The *E. coli* consensus RBS sequence (pBADTHC) (tandem placement 5’ of both *aar* and *adc*) achieved the highest combined hydrocarbon titer, while the calculated (calRBS_aar__*aar* and calRBS_adc__*adc*) and the *R. eutropha nrdD* (tandem placement 5’ of both *aar* and *adc*) RBSs produced about 70% and 30% as much, respectively. The calculated RBSs achieved the most balanced pentadecane:heptadecene ratio. Since changing the pBBR1 origin/*E. coli* consensus RBS sequence combination (pBADTHC) to either mutant pCM271 origin/*E. coli* consensus RBS sequence (pCM271THC) or pBBR1 origin/calculated RBS sequence (pBADTcalRBSHC) combinations did not dramatically reduce combined hydrocarbon titers, but produced a more balanced pentadecane:heptadecene ratio, we constructed plasmid pCM271TcalRBSHC to evaluate the hydrocarbon titer of the mutant pCM271 origin/calculated RBS sequence combination. Surprisingly, pCM271TcalRBSHC achieved a 6-fold improvement in combined hydrocarbon titer (~6 mg/L, Figure 3) relative to pBADTHC, the highest titer construct using previously established *R. eutropha* expression system components [7]. Furthermore, pCM271TcalRBSHC achieved a 100-fold improvement over the lowest production plasmids, pCMTHC and pYIUV5THC (Figure 4).

### Relationship between RFP expression level and hydrocarbon production titer

To visually assess the relationship between toolbox component effects on RFP expression level and hydrocarbon production titer, the pBADT expression cassette (consisting of a pBBR1origin, PBAD promoter, T7 stemloop sequence, and *E. coli* consensus RBS) was used as a normalization point of reference. The reference plasmids pBADTrfp and pBADTHC were normalized to 100% relative RFP intensity and hydrocarbon production titer, respectively. The relative percentages for RFP intensity and hydrocarbon production titer for other expression cassette plasmid pairs are presented in Figure 4. Relative RFP fluorescence intensity appears to only slightly positively correlate linearly with relative hydrocarbon titer.

## Discussion

Our reporter construct results revealed a dynamic range of *rfp* expression levels for the genetic parts in the toolbox (Figure 1) and rapidly identified those parts that are non-functional in *R. eutropha* H16 ∆2303 (Additional file 1: Figure S1). We then investigated the impact of the toolbox on hydrocarbon production titer in *R. eutropha*, and compared the resulting titers with corresponding *rfp* levels across the various expression configurations. In our system, RFP fluorescence intensity weakly correlated with hydrocarbon titer (Figure 4), suggesting that RFP fluorescence intensity is only a modest predictor of hydrocarbon titer, and should not generally be assumed to be a surrogate for pathway genes. This is perhaps not surprising, given that: 1) even RFP and GFP expression may only weakly correlate with each other over identical expression configurations [22], and thus RFP expression may not be a reliable reporter of hydrocarbon production pathway expression; and 2) product titer may not linearly or monotonically relate to pathway expression (*i.e*., more pathway expression does not necessarily translate to higher product titers [23]). While future work will be required to explain the mechanism behind the unexpected 6-fold improvement in combined hydrocarbon titer for pCM271TcalRBSHC over pBADTHC (Figures 3 and 4), we suspect that subtle differences in pathway expression may have serendipitously resulted in substantial titer increases. As such, what is more important than simply maximizing expression of pathway components is the capability to fine tune expression with sufficient granularity to resolve pathway bottlenecks and alleviate toxicity effects. The work reported here specifically contributes to this important capability.

We were surprised to observe that including the pCM271 vector and/or the calculated RBS parts in the hydrocarbon pathway expression construct resulted in balanced levels of pentadecane:heptadecane production in contrast with all other configurations for which the ratio was skewed predominantly to pentadecane (Figures 2 and 3). While the underlying mechanism for the relationship between pentadecane:heptadecane skew on expression configuration remains to be elucidated, it is interesting that using various components of the toolbox developed here affected not only overall product titers, but also product ratios.

Although we did not leverage this capability here, it is worth noting that since plasmid pBADrfp (BBR1 origin, kanamycin resistance) co-propagated stably with pKTrfp (KT origin, chloramphenicol resistance) or pCMrfp (CM62 origin, tetracycline resistance) in *R. eutropha*, it would be possible to engineer a multi-gene metabolic pathway across two plasmid vectors in the same cell (for example, see [24]). Furthermore, promoter cross-induction test results (Table 2) suggest that separate inducible promoters could be used to independently tune the expression of different portions of the pathway. These capabilities will play important future roles in engineering and optimizing more complex metabolic pathways in *R. eutropha*.

Metabolic engineering efforts often focus on a small set of microbial hosts, such as *E. coli* and *Saccharomyces cerevisiae*, simply because there are many established genetic and heterologous gene expression tools available for them. This select group of model microbes, however, may have limited utility for many industrial applications of interest. On the other hand, microbial hosts with metabolic capabilities and growth conditions well suited for specific industrial applications (like *R. eutropha*, which can function as a chemolithoautotroph), but with limited genetic tools, are extremely challenging and time-consuming to metabolically engineer, and developing new genetic tools for specific microbes of interest can be entire research efforts in and of themselves [25]. Here, we have developed and deployed the toolbox for the metabolic engineering of *R. eutropha*. From the outset, we designed our efforts with broad-host range applicability in mind so that we could readily apply the same tools to other microbial hosts of interest. For example, the RSF1010 origin-derived pKT plasmids developed here are able to replicate in a wide range of Gram-negative bacteria (*e.g*., *Enterococci*) as well as the phylogenetically distant cyanobacteria, which are also important hosts of interest for biofuels production [10]. Plasmids within the IncP incompatibility group (including pCM62) and the P_xyls/PM_ promoter system have been demonstrated to function in *Pseudomonas putida*[26,27]. All vectors reported here contain *mob* genes to bolster efficacy across a wide range of bacteria. We envision that the broad-host range toolbox developed here will serve as a turnkey foundation for developing a robust set of metabolic engineering tools for other microbes of interest by simplifying and streamlining the process of screening for functional expression systems that operate within the microbe of interest. Building upon this vision, the toolbox could be exploited to screen metabolic pathway performance across multiple microbial hosts, through the direct transfer of constructs (*e.g*., pCM271TcalRBSHC) to microbes with overlapping functional expression systems. This approach, especially when coupled with no or low leakage inducible promoters (*e.g*., P_BAD_T7, Figure 1B), may be particularly effective for identifying microbial hosts that are tolerant to target or pathway intermediate compounds that are toxic to model microbes such as *E. coli*.

## Conclusions

In this work, we have developed a toolbox for the metabolic engineering of *R. eutropha* H16, comprising six vectors spanning three compatibility groups, four promoter systems responding to three chemical inducers, a T7 5’ mRNA stem-loop structure, and three RBSs. The major contribution of this work is that through increasing genetic regulatory part diversity, we have extended the dynamic range and tunable granularity of gene expression available for *R. eutropha*. We have demonstrated the value of the developed toolbox by increasing combined pentadecane and heptadecene hydrocarbon production titer 6-fold over that achievable with previously available gene expression tools and 100-fold over that achieved by our lowest producing engineered strains. Due to the broad-host range of the selected vectors and mobilized plasmid construction, this toolbox has a great potential to be applied to other microbial hosts for metabolic engineering purposes.

## Materials and methods

### Bacterial cultivation

*R. eutropha* H16 (ATCC 17669), *R. eutropha* H16 ∆2303 [11], *E. coli* DH10B (NEB) and S17 [12] were grown at 30°C in lysogeny broth (LB). Kanamycin (50 mg/L for *E. coli*; 200 mg/L for *R. eutropha*), ampicillin (50 mg/L), chloramphenicol (30 mg/L), tetracycline (10 mg/L) and/or gentamycin (10 mg/L) were added to the medium as appropriate.

### Plasmid construction

With the exceptions of pBADTrfp, pBADTcalRBSrfp, pBADT7Trfp, pBADTnrdDRBSrfp, pXylsTrfp, pKTrfp, pCMrfp, pCM271rfp, pCM273rfp, and pCM291rfp (see Additional file 1), plasmids were assembled with the CPEC or Gibson methods [28,29], and corresponding DNA assembly protocols and DNA oligo primers were designed with j5 and DeviceEditor [30,31]. DNA templates were PCR-amplified with Phusion high-fidelity polymerase (Thermo Scientific). PCR products were gel purified before CPEC or Gibson assembly. The assembled plasmids were either transformed into *E. coli* DH10B, screened by colony PCR [32], sequence validated (Quintara Biosciences), and then transformed into *E. coli* S17 or directly transformed into *R. eutropha* H16 ∆2303, screened by colony PCR, and then sequence validated. Plasmids were then transconjugated from *E. coli* S17 into *R. eutropha* H16 ∆2303 as previously described [12].

### Strain and plasmid availability

The strains and plasmids used in this study are listed in Table 1. All strains and plasmids developed here, along with their associated information (*e.g*., annotated GenBank-format sequence files, sequence validation trace files, DeviceEditor design files, and j5 design output files), have been deposited in the public instance of the JBEI Registry [33] (https://public-registry.jbei.org; entries JPUB_001171-JPUB_001230) and are physically available from the authors and/or addgene (http://www.addgene.org) upon request.

### RFP fluorescence assay

To measure the fluorescence intensity of RFP (monomeric mRFP1, maturation < 1 hour ) [34] expressed from each type of plasmid vector, single colonies were picked and inoculated into LB seed-culture tubes supplemented with kanamycin, chloramphenicol, gentamycin, or tetracycline, as appropriate. 100 μL of each overnight seed culture was inoculated into a fresh 5 mL LB culture tube supplemented with the appropriate antibiotic and inducer (IPTG, L-arabinose, *m*-toluic acid, or tetracycline) and grown at 30°C, 200 rpm for 48 hours. 100 μL of each cell culture tube was then added to a separate well in a 96-well clear-bottom plate (Corning: No. 3631) and RFP fluorescence was measured with a Safire (Tecan) microplate reader using an excitation wavelength of 585 nm and an emission wavelength of 620 nm. OD_600_ was also measured for each well immediately thereafter to calculate the RFP fluorescence intensity/OD_600_ ratio reported for this assay.

### Plasmid copy number assay

Single colonies (serving as templates) were picked, suspended in water, and then boiled for 10 minutes. Primers (pcmF and pcmR, phaZF and phaZR; Additional file 1) to amplify 400 to 500 bp fragments of *rfp* and *phaZ* respectively, were designed with Clone Manager 8.0. qPCR reactions were performed with a StepOnePlus Real-Time PCR System (Life Technologies) with Maxima SYBR Green/Fluorescein qPCR Master Mix (Thermo Scientific) as recommended by the manufacturers. Three biological replicates, with two technical replicates each, were performed for each plasmid type. Absolute plasmid copy numbers were determined using the CT difference between the plasmid-borne *rfp* gene and the single copy chromosomal *phaZ* gene.

### Inducer dose response assay

For each plasmid type, 10 μL of overnight LB cell culture was inoculated into each of 4 separate 24-well clear-bottom plate wells containing 1 mL fresh LB supplemented with a varying concentration of the appropriate inducer. These culture plates were then grown in a Pro200 (Tecan) microplate reader at 30°C, 37 rpm, for 72 hours. OD_600_ and RFP fluorescence intensity, using an excitation wavelength of 585 nm and an emission wavelength of 620 nm, were measured after 48 hours.

### Hydrocarbon production assay

Single colonies were picked and inoculated into 10 mL fresh LB glass culture tubes and grown at 30°C for 15 hours. The appropriate inducer was then added to each culture tube (final concentration: 1 mM IPTG, 0.1% L-arabinose, or 1 mM *m*-toluic acid). 1 mL decane (Sigma, 99% purity) was then immediately added to each 10 mL culture tube to extract hydrocarbons and other metabolites. 72 hours after induction, 100 μL of each decane overlay was removed for direct gas chromatography–mass spectrometry (GC/MS) analysis. Electron ionization (EI) GC/MS analyses were performed with a model 7890A GC quadrupole mass spectrometer (Agilent) with a DB-5 fused silica capillary column (J & W Scientific, 30-m length, 0.25-mm inner diameter, 0.25-μm film thickness) coupled to a HP 5975C mass selective detector. 1 μL injections were performed by an Agilent model 7683B autosampler. The GC oven was typically programmed to ramp from 40°C (held for 3 minutes) to 300°C at 15°C/min and then held for 5 minutes. The injection port temperature was 250°C, and the transfer line temperature was 280°C. The carrier gas, ultra-high purity helium, flowed at a constant rate of 1 mL/min. Injections were splitless, with the split turned on after 0.5 minutes. For full-scan data acquisition, the MS typically scanned from 50 to 600 atomic mass units at a rate of 2.7 scans per second. One of the major products, pentadecane (15:0), was quantified with *m*/*z* 57 area, while the other major product, heptadecene (17:1), was quantified with *m*/*z* 83 area of authentic standards (Sigma, 99% purity).

## Abbreviations

R. eutropha: Ralstonia eutropha; PHB: Polyhydroxybutyrate; RBS: Ribosomal binding site; LB: Lysogeny broth.

## Competing interests

The authors declare that they have no competing interests.

## Authors’ contributions

CB designed and carried out this work, and drafted the manuscript. PS participated in experimental design, and molecular genetic studies and other experimental aspects of this work. JM participated in experimental design and strain construction. YY participated in genetic studies. SRC, HRB, and SWS supervised the research and edited the manuscript. NJH supervised the research, and wrote and edited the manuscript. All authors read and approved the final version of the manuscript.

## Supplementary Material

Additional file 1 Supplementary Materials and Methods. Click here for file
