# Supplementary material for: Development of a broad-host synthetic biology toolbox for ralstonia eutropha and its application to engineering hydrocarbon biofuel production
Source: Microb Cell Fact. 2013 Nov 13;12:107. doi: 10.1186/1475-2859-12-107 (PMC3831590; doi:10.1186/1475-2859-12-107)
Supplement: Additional file 1 — Supplementary Materials and Methods. [file 1475-2859-12-107-S1.docx]

**Supplemental Materials**

**Materials and Methods**

**Plasmid construction**

pBADTrfp. pBADrfp backbone was digested with PstI and EcoRI, and ligated with the similarly digested PCR product resulting from amplifying the region upstream of *rfp* in pBADrfp with primer pair KanF and T7SLR (Table S1), which contains the T7 stem loop sequence.

pBADTcalRBSrfp. pBADTrfp was PCR amplified with primer pair pBADTrfp5242F, containing the calculated RBS sequence, and pBADTrfp5212R. The PCR product was phosphorylated with T4 polynucleotide kinase and blunt auto-ligated to yield pBADTcalRBSrfp.

pBADT7Trfp. The T7 RNA polymerase gene from *E. coli* DH1 (DE3) [[1](#_ENREF_1)] was PCR amplified with primer pair T7polyF and T7polySLR, which contains the T7 stem loop sequence. pBADrfp backbone was PCR amplified with primer pair pBADTrfp5193F and pBADTrfp5180R. Both PCR products were digested with EcoRI and BglII and ligated together to yield pBADT7Trfp.

pBADTnrdDRBSrfp. pBADrfp backbone was PCR amplified with primer pair pBADTrfp2576F and pBADTrfp731R. The *rfp* gene in pBADrfp was PCR amplified with primer pBbS2c-RFP742rbsnrdDF, containing the RBS sequence of *R. eutropha* *nrdD* gene, and pBbS2c-RFP1681R. The two PCR products were phosphorylated with T4 polynucleotide kinase and ligated together to obtain pBADTnrdDRBSrfp. Colonies were screened with enzyme digestion to identify clones with correct insert directionality.

pXylsTrfp. A Xyls/P_m_ gene fragment was designed with Gene Designer (DNA 2.0) with *R. eutropha* codon preference, flanked with AatII and EcoRI sites, and synthesized by Genscript (New Jersey, US). The synthesized DNA fragment was digested with AatII and EcoRI and ligated into similarly digested pBADTrfp to obtain pXylsTrfp.

pKTrfp. pBbE8c-RFP template was amplified with primers cmrfpF and cmrfpR to obtain the *cmR*-pBAD*-rfp* cassette. NsiI and NaeI restriction sites embedded in the primers were utilized to insert these fragments into PvuII and PstI treated pKT230. NsiI is compatible with PstI; and NaeI is compatible with PvuII.

pCMrfp. Primers pCM62F and pCM62R were used to amplify the backbone of pCM62. Restriction sites AvrII and SpeI were embedded in the two primers, respectively. The PCR product was digested with AvrII and SpeI and ligated to the corresponding pBAD-rfp fragment from pBbA8a-RFP, treated with the same enzymes.

pCM271rfp, pCM273rfp, and pCM291rfp. These plasmids were generated by Quick Change Mutagenesis [[2](#_ENREF_2)] of pCM62rfp. The primers used to introduce the point mutations, pCM271F and pCM271R, pCM273F and pCM273R, pCM291F and pCM291R, are listed in Table S1.

**References**

1. Bond-Watts BB, Bellerose RJ, Chang MCY: **Enzyme mechanism as a kinetic control element for designing synthetic biofuel pathways.** *Nature Chemical Biology* 2011, **7:**222-227.

2. Wang WY, Malcolm BA: **Two-stage PCR protocol allowing introduction of multiple mutations, deletions and insertions using QuikChange (TM) site-directed mutagenesis.** *Biotechniques* 1999, **26:**680-682.

**Table S1.** Primers used in this study.

| Primer Name | Primer sequence |
| --- | --- |
| KanF^a^ | cttcagtgacaacgtcgagcac |
| T7SLR | ttt**gaattc**caaaattatttctagagggaaaccgttgtggtctccctatggagaaacagtagagagttgcgat |
| T7polyF | cacacca**gaattc**ttgatggcgtcgggatctg |
| T7polySLR | ttt**agatct**caaaattatttctagagggaaaccgttgtggtctcctgcaaaaagaacaagtagcttgtattccctgggatccggagtcgtattg |
| pBADTrfp5193F^a^ | ccctctagaataattttgg |
| pBADTrfp5180R^a^ | tctccctatggagaaac |
| pBADTrfp5242F | gaggcccggaacagaagaaggagtacacaatacatatggcgagtagc |
| pBADTrfp5212R | ccaaaattatttctagagggaaac |
| pBADTrfp2576F | atattttatctgttgtttgtcggtgaac |
| pBADTrfp731R | atatgaattcttttctctatcactgatagg |
| pBbS2c-RFP742rbsnrdDF | gccgggagaatgtatggcgagtagcgaagacgttatc |
| pBbS2c-RFP1681R | aaatagcgctttcagccggcaaacc |
| cmrfpF | aaaa**atgcat**actagtgcttggattctcaccaa |
| cmrfpR | aaaa**gccggc**cctaggtataaacgcagaaaggcc |
| pCM62F | aaaa**cctagg**tcgtgatacgcctatttttataggttaatg |
| pCM62R | aaaa**actagt**cagaagtggtcagcttggct |
| pCM271F | gtgtcgctgctgcactgcttccgcgtcctggaccgtgg |
| pCM271R | ccacggtccaggacgcggaagcagtgcagcagcgacac |
| pCM273F | gtgtcgctgctgcaccgcttctgcgtcctggaccgtgg |
| pCM273R | ccacggtccaqggacgcagaagcggtgcagcagcgacac |
| pCM291F | ggtcctgatcgacgagggaatcgtcgtgctgtttgc |
| pCM291R | gcaaacagcacgacgattccctggtcgatcaggacc |
| pcmF | acgaaggtacccagaccgctaaac |
| pcmR | tcacctttcagagcaccgtcttcc |
| phaZF | tccgacgcaatctggtttaccc |
| phaZR | gccaaaggcgatctgaaactcc |

Embedded restriction sites indicated with **bold** lettering.

^a^Restriction site internal to PCR product, and not embedded within the primer itself.

**Figures**

Promoter:

P_lacUV5lacI_ P_lacUV5_ P_lacUV5lacIlacY_

Fluorescence intensity/OD_600_

**Figure S1.** Fluorescence intensity output of plasmids with promoters derived from P_lacUV5_.

Hydrocarbons

µg/L

**Figure S2.** Hydrocarbon production of expression plasmids with and without the T7 stemloop. pBADTHC carries the T7 stem loop sequence; pBADHC does not.
